# Expression of a phage RecET recombinase system improves detection of shorter DNA fragments by an *Acinetobacter baylyi* ADP-ISx antibiotic gene biosensor strain

**DOI:** 10.17912/micropub.biology.002084

**Published:** 2026-04-21

**Authors:** Nicholas Rhyan, Issac S. Koshy, Ryan Dinh, Anthony J. VanDieren, Dennis M. Mishler, Jeffrey E. Barrick

**Affiliations:** 1 Freshman Research Initiative, The University of Texas at Austin, Austin, TX, US; 2 Department of Molecular Biosciences, The University of Texas at Austin, Austin, TX, US; 3 Department of Microbiology, Genetics, & Immunology, Michigan State University, East Lansing, MI, US; 4 Department of Entomology, Michigan State University, East Lansing, MI, US

## Abstract

Naturally competent bacteria have been engineered into sequence-specific biosensors for environmental DNA, but low recombination rates limit detection of small DNA fragments. Phage recombinases are often used to increase the efficiency of integrating DNA constructs with short homology arms into bacterial genomes. We show that expression of a RecET homolog from an *Acinetobacter baumannii* prophage improves *Acinetobacter baylyi* ADP1-ISx biosensor detection of 120- and 200-bp fragments of an antibiotic resistance gene by approximately 10- to 20-fold. This strategy can potentially enable more sensitive detection of target sequences in highly degraded DNA from environmental samples by cell-based bacterial biosensors.

**Figure 1. Expression of RecET-Ab improves detection of short DNA fragments from an antibiotic resistance gene by an engineered A. baylyi biosensor strain:** ( **a** ) DNA sensing by *A. baylyi* ADP1-ISx *nptII* Δ10. The chromosome of this kanamycin-sensitive (Kan ^s^ ) biosensor strain contains a copy of the *nptII* resistance gene that has been inactivated by a 10-bp deletion. DNA fragments matching the intact *nptII* gene are detected when they are imported by cells and recombine to repair the copy in the chromosome, resulting in kanamycin resistant (Kan ^r^ ) cells. ( **b** ) Donor DNA plasmids. A series of pUC plasmids were constructed to provide intact *nptII* fragments of different sizes centered on the deletion in the sensor construct. These plasmids do not replicate in *A. baylyi* . ( **c** ) DNA detection assay. Cultures of biosensor cells, either with no plasmid or expressing the RecET-Ab phage recombinase from plasmid pAT02, were incubated with donor plasmid DNA to test for differences in transformation frequencies. ( **d** ) Dependence of DNA detection on RecET-Ab expression as a function of the size of the intact *nptII* homology region in the donor DNA plasmid. Error bars are 95% confidence intervals estimated using a negative binomial model (see Methods). Statistically significant differences between the RecET-Ab and no plasmid strains were determined using Bonferroni-corrected likelihood-ratio tests (n.s., not significant; *, *p* < 0.05; **, *p* < 0.01). &nbsp;

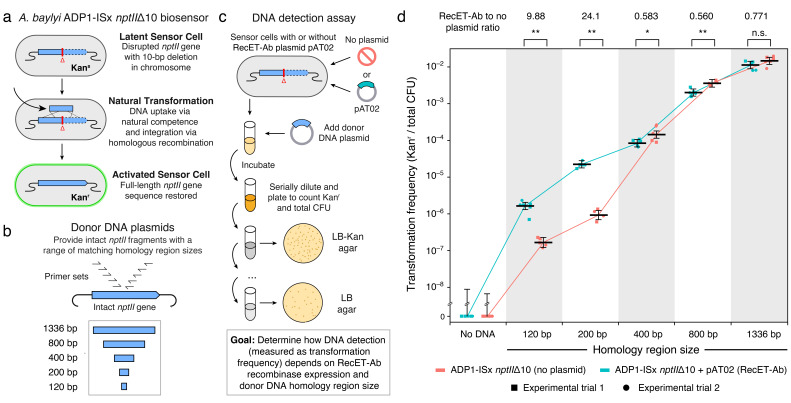



## Description

Recently, naturally competent bacteria have been engineered into cell-based biosensors for detecting specific DNA sequences in the human genome (Nou and Voigt 2024), tumor cells (Cooper, et al. 2023), ancient DNA samples (Overballe-Petersen, et al. 2013), and microbial pathogens (Cheng, et al. 2023; Chuong, et al. 2025). Most of these studies use repair of an inactivated antibiotic resistance gene in the genome of the sensor strain for DNA detection or for testing DNA sensing parameters. DNA detection is limited by the efficiency with which environmental DNA is imported into cells and integrated into the genome. Native RecA-mediated homologous recombination supports efficient integration of DNA fragments with >1000 bp matching a sensor sequence, but detection rates precipitously decline for shorter DNA fragments (Simpson, et al. 2006; Overballe-Petersen, et al. 2013). Expression of phage recombinases that can efficiently operate on homology regions as short as 50 bp could potentially mitigate this limitation. For example, the Rac RecET and λ Red recombinase systems are commonly used for this purpose in *Escherichia coli* to improve the efficiency of genome engineering procedures (Sharan, et al. 2009; Wannier, et al. 2021).&nbsp;

*Acinetobacter baylyi* is a popular bacterial chassis for metabolic engineering and synthetic biology because of its natural competence for DNA uptake (Metzgar, et al. 2004; Elliott and Neidle 2011; Biggs, et al. 2020; Santala and Santala 2021). Multiple research groups have leveraged this ability to create *A. baylyi* DNA biosensors (Overballe-Petersen, et al. 2013; Cooper, et al. 2023; Chuong, et al. 2025). Other researchers have shown that expression of a RecET homolog identified in an *Acinetobacter baumannii* prophage (RecET-Ab) allowed them to integrate electroporated DNA fragments with shorter homology arms into the chromosome of this *Acinetobacter* species (Tucker, et al. 2014). Inspired by these results, we hypothesized that the RecET-Ab recombinase could improve detection of smaller DNA fragments by *A. baylyi* biosensor cells, given the close evolutionary relationship between these organisms.

We evaluated how homology region size and RecET-Ab expression influenced DNA detection by a previously constructed *A. baylyi* ADP1-ISx *nptII* Δ10 biosensor strain (Chuong, et al. 2025). *A. baylyi* ADP1-ISx is an engineered transposon-free variant of ADP1 that exhibits improved transformation frequencies (Suárez, et al. 2017). The *nptII* gene encodes the enzyme neomycin phosphotransferase II, which confers resistance to the antibiotic kanamycin. The sensor strain encodes an *nptII* variant inactivated by a 10-bp deletion, *nptII* Δ10, in its chromosome. In this system, uptake and recombination of environmental DNA containing all or a portion of an intact *nptII* gene restores the chromosomal sequence ( **Fig. 1a** ). Cells that have detected *nptII* DNA become kanamycin resistant, and there is no detectable background rate of kanamycin resistance from spontaneous mutations (Chuong, et al. 2025).

For our study, we constructed a series of pUC-based donor DNA plasmids containing 120, 200, 400, 800, or 1336 bp of the intact *nptII* gene centered on the 10-bp deletion in the sensor construct by cloning them in *E. coli* cells ( **Fig. 1b** ). pUC and other ColE1 origin plasmids do not stably replicate in *A. baylyi* (Hunger, et al. 1990; Palmen, et al. 1993), so these plasmids only serve as a source of incoming DNA for integration into the chromosome. *A. baylyi* ADP1-ISx *nptII* Δ10 sensor cells were transformed with the pAT02 plasmid, which has an RSF1010 origin of replication and encodes the RecET-Ab system under control of an IPTG-inducible promoter regulated by LacI expressed from the plasmid (Tucker, at al. 2014). RSF1010 plasmids and LacI-regulated promoters have been shown to function in *A. baylyi* in prior work (Murin, et al. 2012; Geng, et al. 2019; Biggs, et al. 2020), and we found that pAT02 stably replicated and was maintained when we included carbenicillin in media to select for the plasmid.&nbsp;

Because we were unsure to what extent leaky expression of RecET-Ab from the pAT02 plasmid in the absence of IPTG would interfere with our results, we compared DNA detection by ADP1-ISx *nptII* Δ10 cells with no plasmid to those with pAT02 and IPTG-induction. Successful uptake and homologous recombination restore kanamycin resistance, allowing transformation frequencies to be measured by plating dilutions on selective and nonselective agar and counting colony-forming units (CFUs) ( **Fig. 1c** ). We found that RecET-Ab expression greatly improved DNA detection (measured as transformation frequency) for the 120- and 200-bp homology region donor DNA plasmids ( **Fig. 1d** ), by factors of 9.88 and 24.1, respectively. For donor DNA plasmids with homology regions of 400 or 800 bp, RecET-Ab caused a slight reduction in transformation frequency, reducing it by about 50%. This modest inhibition of DNA sensing might result from the presence of the plasmid and/or expression of RecET-Ab interfering with native RecA-mediated homologous recombination or having other off-target effects.

We demonstrated that expression of a RecET recombinase identified in an *A. baumannii* prophage can improve *A. baylyi* detection of short DNA fragments by an order of magnitude or more. With further optimization, this approach could be a key component to making more sensitive bacterial biosensors suitable for detecting degraded extracellular DNA in real-world environments (Blackman, et al. 2024). RecET-Ab expression also has the potential to make other *A. baylyi* genome engineering procedures more efficient, such as transforming mutagenized PCR products or synthetic DNA fragments to introduce genetic diversity at a specific locus (Kok, et al. 1997). Finally, our results suggest that expressing prophage-derived recombinases, such as those native to *B. subtilis* or its relatives (Liu, et al 2023; Xue, et al. 2023) **,** could similarly improve detection of shorter DNA fragments in other biosensor chassis.

&nbsp;

## Methods

**Media and culture conditions**

*A. baylyi* and *E. coli* were cultured in LB in glass test tubes with shaking or plated on LB-agar in plastic petri dishes. Media were supplemented with 60 µg/mL spectinomycin (Spt), 50 μg/mL kanamycin (Kan), or 100 μg/mL carbenicillin (Crb) where indicated. *A. baylyi* and *E. coli* were incubated at 30°C or 37°C for growth, respectively. Cultures were stored as frozen stocks at –80°C after adding glycerol to 10-20% (v/v) as cryoprotectant.

**Donor DNA plasmid assembly&nbsp;**

We used Phusion polymerase to PCR amplify a series of fragments of the *nptII* gene with defined lengths (120, 200, 400, 800, and 1336 bp) from plasmid pKD13 (Datsenko and Wanner 2000). Primers were designed to create amplicons centered on the 10-bp mutation in the inactivated *nptII* gene in the sensor strain with flanking BsmBI sites for cloning. We checked PCR product sizes on agarose gels. Then, after purification using the Monarch DNA Gel Extraction Kit, we cloned them into pBTK1149, a GFP-dropout entry vector with a pUC origin and spectinomycin resistance marker compatible with standard YTK/BTK workflows (Lee, et al. 2015; Leonard, et al. 2018), using BsmBI Golden Gate assembly. All five resulting plasmids were transformed into *Escherichia coli* DH5α and purified using a QIAprep Spin Miniprep Kit. We named these plasmids pDonor-nptII-#bp where # is the length of the *nptII* gene fragment cloned into each one. Plasmid stock concentrations were measured using a Qubit fluorometer (ThermoFisher). Full plasmid sequences determined by nanopore sequencing (Plasmidsaurus) are provided in **Extended Data 1** .

**Recombinase plasmid transformation**

We used natural transformation to deliver the pAT02 recombinase plasmid to ADP1-ISx *nptII* Δ10 sensor cells. The transformation reaction consisted of 500 µL LB, 35 µL of sensor strain overnight culture, and 234 ng of purified pAT02 plasmid DNA in 10 µL. It was incubated for 6 h at 30º C with shaking then plated on LB-Crb agar to select for cells that acquired the plasmid. We grew overnight cultures of selected colonies, created frozen stocks, and ran DNA purified using a QIAprep Spin Miniprep Kit on an agarose gel to verify that they contained the plasmid.

**Transformation assays**

Cultures of *A. baylyi* ADP1-ISx *nptII* Δ10 with and without plasmid pAT02 were grown overnight in LB from freezer stocks, supplementing with Crb for the plasmid-containing strain. Two master mixes were then prepared, each beginning with 10 mL of LB in a 15 mL conical tube. The master mix of the strain with pAT02 received an additional 10 µL of Crb and 21.4 µL of an IPTG stock that supplied a final IPTG concentration of 2 mM. To each master mix, 700 µL of the corresponding overnight culture was added (equivalent to 35 µL of culture per 500 µL of LB). After gentle vortexing, 535 µL of each master mix was dispensed into eighteen 1.7 mL microcentrifuge tubes. For each homology length, we added 10 pmol of donor plasmid DNA to three of these tubes for each strain. Plasmid stock solutions were pre-diluted such that at least 10 µL was added to ensure accurate pipetting. Three tubes for each strain were negative controls with no DNA added. All transformation mixes were transferred to test tubes and incubated for 6 h at 30°C with shaking. Following incubation, transformations were transferred to new microcentrifuge tubes, and serial dilutions were prepared in sterile saline by successive 1:10 steps until reaching 1:100,000, followed by a final 1:5 dilution to obtain a 1:500,000 dilution. For each transformation assay, 50 µL of an appropriate dilution was plated on selective LB-Kan agar to count transformant CFUs, and 50 µL of the 1:500,000 dilution was plated on nonselective LB agar to count total CFUs. For selective plating, the dilutions to use were calculated based on preliminary results to achieve 30-300 CFUs. Plates were incubated at 30°C for 24 hours until colonies were countable. Two replicate trials of the entire experiment were conducted at different times. Colony counts for all transformation assays are provided in **Extended Data 2** .

**Statistical analysis**

To calculate transformation frequencies, CFUs on selective plates (LB-Kan) were compared to CFUs on nonselective plates (LB). For samples transformed with plasmid DNA, a negative binomial generalized linear model was fit to the CFU count response. The model’s predictors were a factor with levels for each condition (combination of strain and DNA homology region size tested) and a factor for selective versus nonselective plate. Plating dilution factors were incorporated through offsets. We used this model to estimate one overall size parameter for the data and the maximum likelihood transformation frequency and 95% confidence intervals on this estimate for each condition. The statistical significance of a frequency difference at a given homology length between the no plasmid and pAT02 strains was evaluated by comparing negative binomial models with and without this additional factor using likelihood-ratio tests. The size parameters for these fits were fixed at the value estimated from the overall model. Adjusted *p* -values for the five comparisons at the different homology lengths (100, 200, 400, 800, and 1336 base pairs) were calculated using the Bonferroni correction for multiple testing.&nbsp;

For the no DNA controls where all CFU counts on selective plates were zero, we used Monte Carlo sampling to estimate an upper 95% confidence limit on the transformation frequency. Colony count rates on nonselective plates were generated by sampling from the distribution fit by negative binomial regression with the size parameter fixed at that determined for samples with non-zero counts. Colony count rates on selective plates were generated from the distribution of the true mean conditional on the observation of zero counts under a negative binomial model with the same fixed size parameter using inverse transform sampling. The empirical 97.5% quantile on the ratios of one million sampled selective and nonselective rates was used to estimate a final upper 95% confidence limit consistent with the observation of zero counts. The R script used for data analysis and visualization is provided as **Extended Data 3** .&nbsp;

## Reagents

**Table:** 

**Reagent**	**Supplier/Catalog # or Recipe**
*Acinetobacter baylyi* ADP1-ISx *nptII* Δ10	Chuong, et al. 2025
*Escherichia coli* DH5ɑ	ThermoFisher (18265017)
Plasmid pAT02	Tucker, et al. 2019
Plasmid pKD13	*E. coli* Genetic Resource Center (CGSC#: 7633)
Plasmid pBTK1149	This study
Plasmid pDonor-nptII-120-bp	This study
Plasmid pDonor-nptII-200-bp	This study
Plasmid pDonor-nptII-400-bp	This study
Plasmid pDonor-nptII-800-bp	This study
Plasmid pDonor-nptII-1336-bp	This study
QIAprep Spin Miniprep Kit	QIAGEN (27104)
Monarch DNA Gel Extraction Kit	New England Biolabs (T1020)
Phusion High-Fidelity DNA Polymerase	New England Biolabs (M0530L)
NEBridge Golden Gate Assembly Kit (BsmBI-v2)&nbsp;	New England Biolabs (E1602L)
Spectinomycin Dihydrochloride Pentahydrate (Spt)	GoldBio (S-140-5)
Kanamycin Monosulfate (Kan)&nbsp;	GoldBio (K-120-5)
Carbenicillin (Disodium) (Crb)&nbsp;	GoldBio (C-103-5)
Isopropyl β-D-1-thiogalactopyranoside (IPTG)	GoldBio (I2481C)
Lysogeny Broth (LB)&nbsp;	For 1L: 10 g Tryptone, 5 g Yeast Extract, 10 g Sodium Chloride. Autoclave to sterilize.
LB Agar&nbsp;	For 1L: 10 g Tryptone, 5 g Yeast Extract, 10 g Sodium Chloride, 15 g&nbsp; Agar. Autoclave to sterilize.
Sterile Saline	For 1L: 8.5 g NaCl. Autoclave to sterilize.
